# Measurement of gap between abutment and fixture in dental conical connection implants. A focused ion beam SEM observation

**DOI:** 10.4317/medoral.23281

**Published:** 2020-06-10

**Authors:** Fabio Carnovale, Romeo Patini, David Penarrocha, Maurizio Muzzi, Roberto Pistilli, Luigi Canullo

**Affiliations:** 1 Private practice, Rome, Italy; 2 Catholic University, Rome, Italy; 3 University of Valencia, Valencia, Spain; 4 University of Rome 3, Rome, Italy

## Abstract

**Background:**

The aim of the authors was to examine the abutment-fixture interface in Morse-type conical implants in order to verify gaps at this level using a new microscopical approach.

**Material and Methods:**

In this *in vitro* study, 20 abutment-fixture complexes were prepared by sectioning (longitudinal and cross-sectional to the long axis) with a microtome and then with a focused ion beam (FIB). This is a micrometric machine tool that uses gallium ions to abrade circumscribed areas to dig deeper into the cuts obtained with the microtome in order to eliminate cut-induced artifacts. This is because the FIB abrasion is practically free from artifacts, which are normally generated by the action of the microtome blades or other techniques. Samples were then observed by scanning electron microscopy (SEM).

**Results:**

The observation of the abraded parts with the FIB permitted measurement of the real gap between the implant-abutment components. A variable amount of gap was retrieved (from 0 to 3 μm) by the observations, confirming the non-hermetic nature of the connection. It has to be pointed out that in approximately 65% of cases, the gap accounted for less than 1 μm.

**Conclusions:**

The reported data confirmed that the analyzed connection system allowed for minimal gap. However, from the evidence of the present analysis, it cannot be assumed that the 2 parts of a Morse-type conical implant are fused in 1 piece, which would create a perfectly matched hermetic connection.

** Key words:**Dental abutment, dental implant, focused ion beam SEM, implant-abutment connection, morse taper, microbial leakage.

## Introduction

It has been established, since the dawn of modern implantology that, in order to achieve an effective implant osseointegration, it is necessary to delay the load of the dental implant, from the time of surgery until the application of the prosthesis. This allows the bone cells to stabilize around the implant surface over a time period that is sufficient to guarantee the growth of the osteocytes in contact with it (1).

Implants normally consist of 2 parts: the fixture, which is inserted into the bone structure at the time of surgery and the abutment, which is the external portion applied after several weeks. However, even in cases of immediate loading, it is almost always necessary to separate the submerged portion and the emergence profile to compensate for the various angles between the implant and the dental crown or other kinds of prostheses. Such procedures can cause several mechanical and biological issues.

The microbiological issues, in particular, are mostly related to the microinfiltration of bacteria between the 2 metal pieces that can be connected to each other in various ways. In most cases, the 2 parts are held together by a screw, which tightens the 2 components. The coupling of the 2 mechanical parts, although precise and tight, is still subject to bacterial infiltration and proliferation, which can give rise to inflammation of the surrounding tissues, possibly leading to the loss of osseointegration (2).

Many attempts have been made to address these issues by varying the connection between the submerged portion and the emergence profile.

First, the connection has been modified in order to avoid movement between the parts due to mastication and swallowing forces. These movements, other than facilitating the separation between the parts and fracture of the connecting screw, also allow abundant penetration of biological material. The cemented connections may have a lower bacterial penetration rate than the screwed ones but present the problem of material residues in the critical area at the bone level (3,4).

A conical shape, as opposed to flat surfaces, can be considered less subject to movement, even in the long-term (5).

The platform-switching connection, as opposed to the platform-matching connection (obtained mostly with male-female conical parts) was developed to reduce as much as possible the gap between the components. Preliminary and systematic studies have shown this conformation of the abutment-fixture interface to provide a good biological response (6).

The most critical area for bacterial growth, which is considered the main cause of bone resorption in the coronal part of the implant, is the junction between the abutment and the fixture. This junction in bone-level implants is more problematic than in tissue-level or single-body implants (7).

So far, one of the safest implant abutment connection is represented by the conical connection, especially the Morse taper. This conFiguration, other than in dentistry, it is also used in other medical fields, such as orthopedics, and is currently regulated by the Italian edition of the International Organization for Standardization (UNI ISO 296) that certifies its effectiveness (8).

At a microscopic level, the 2 components "interpenetrate" into each other to avoid detachment, presenting a small gap which is supposed to limit the bacterial penetration and limit mechanical instability under loading (2,9-12).

Studies using SEM provided an observation of the surfaces involved, in this case, the implant-abutment junction. The gap between the abutment and the fixture could be evaluated more in-depth with various methods: by cutting with the appropriate microtomes using diamond blades or by abrasion with burs or electroerosion. However, these techniques have the limitation of producing artifacts connected to the mechanical action of the cutting tools. The metal parts are irregular and the real gap cannot effectively be measured and is often invisible.

For this reason, the authors of the present study decided to use the Helios NanoLab DualBeam focused ion beam (FIB) scanning electron microscope (Thermo Fisher Scientific, Waltham, MA, United States), a method of abrasion that is considered to be extremely accurate and almost free of artifacts. In fact, through the microerosion of a gallium ion beam, this device removes the most superficial part of the section and its debris, giving a true image of the gap between the abutment and fixture in dental implants.

Given this, the aim of the present study was to examine the abutment-fixture interface in Morse-type conical implants. The null hypothesis was to find a microleakage.

## Material and Methods

In this *in vitro* study, 20 size 4, 1-mm implants with pure, Morse-type conical connections (Exacone, Leone, Italy) were used in association with 20 abutments for the same implant system.

These complexes were provided with a slot to accommodate the Morse-type abutment with a 3° convergence and a hexahedron in the lower part to avoid displacement and ensure the correct positioning. The application of the abutment consists exclusively in positioning it in the slot and beating 5 strikes with a special tool provided by the manufacturer, taking care to apply the force axially to the fixture.

- Sample Preparation and Observation

After applying the abutment to the fixture following the manufacturer’s protocol, the samples were sectioned by microtome with water-cooling.

Sections were made longitudinally on 10 samples and transversely on other 10 samples.

**Figure 1:**
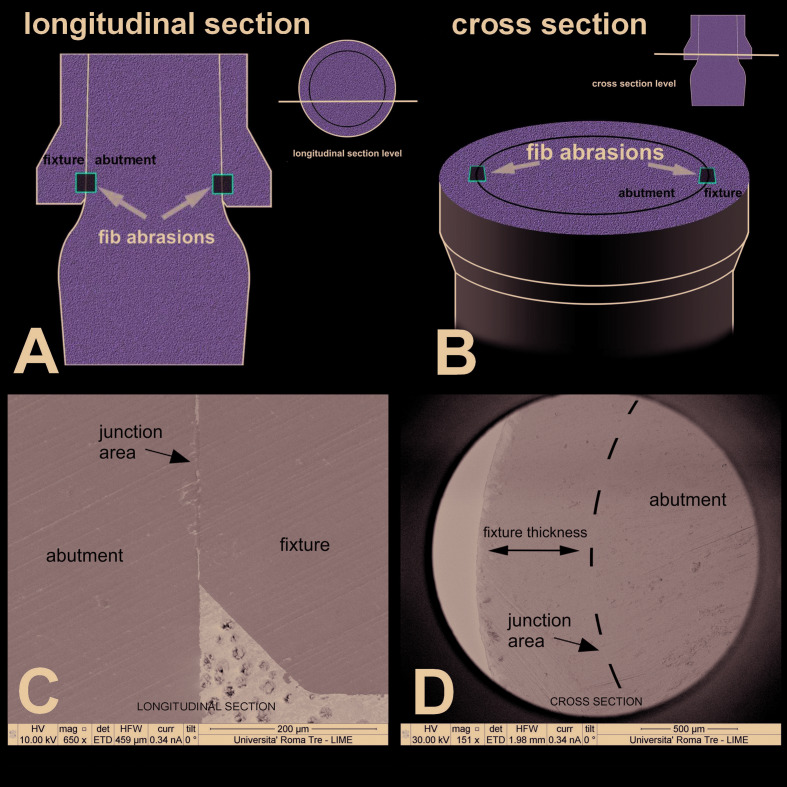
A - Longitudinal section drawing. After applying the abutment, the sample was sectioned longitudinally at 0.5 mm from the center in order to avoid losing mechanical retention. Subsequently FIB abrasions were produced in the areas indicated on both sides. FIB, focused ion beam; B - Cross-sectional drawing. In this case, the cut was made below the contact area between the abutment and the implant collar (platform switching); C - Longitudinal section at low magnification; D - Cross section at low magnification.

**Figure 2:**
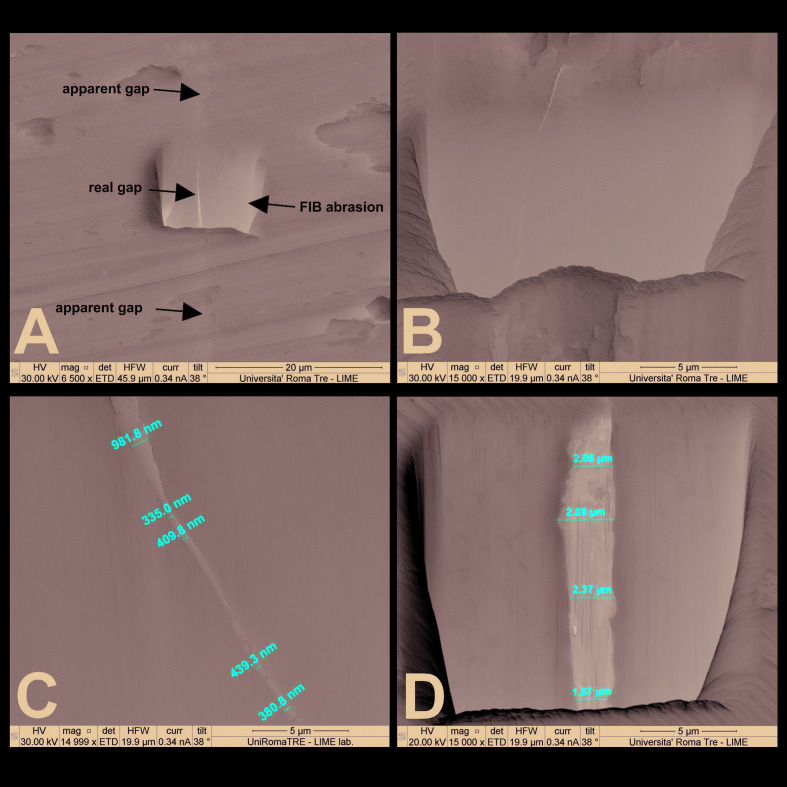
A - The real gap between the parts exposed by FIB abrasion. FIB, focused ion beam; B - Where the parts are in contact, the gap is almost undetectable; C, D - Gap measurement of Morse cone abutment in a longitudinal section.

All samples were further observed during SEM after being subjected to FIB abrasions (at least 2: 1 each side of the longitudinal sections and 2 for the opposite sides of the cross-sections Fig. 1) by the device at 52° and at a depth of 5 µm in order to eliminate the deformed part from the cut. FIB abrasions were made to reveal the real gap, which was below the rough cut made with the microtome. Fig. 2 shows how the FIB works: the gallium ion beam removes the most superficial layer of the dissected titanium, in a very fine way and almost free of artifacts, eliminating the deformations of the cutter and exposing the real gap between the parts.

In total, 10 transverse cuts were made in the implant with the abutment applied, 1-mm below the area where the abutment enters the hollow part of the fixture, and 10 longitudinal cuts were made near the center of the implant 0.5-mm before the middle. This was done to avoid the detachment of the parts through lateral retention.

- Statistical analysis

A descriptive analysis of the data was made. Mean values and standard deviations (SD) were calculated to test the gap.

## Results

The abrasion made by the FIB showed a mean value in microns SD of 0.97 0.21 for cross-sectional cuts and 1.23 0.49 for longitudinal sectional cuts.

**Figure 3:**
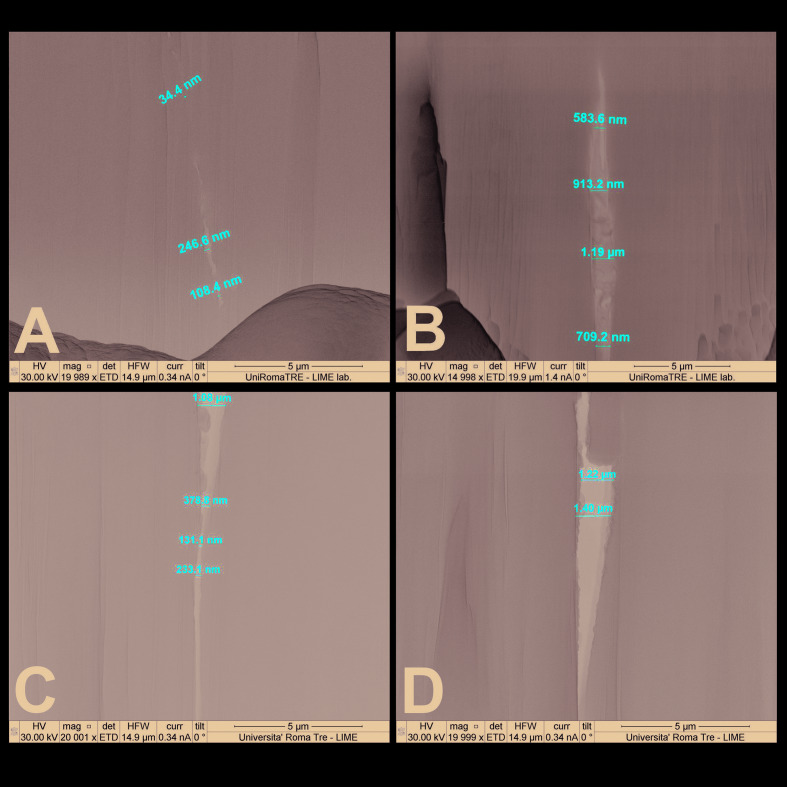
A - Gap measurement of Morse cone abutment in a longitudinal section. B, C, D - Gap measurement of Morse cone abutment in a cross-section.

The context of the FIB abrasion is depicted in Fig. 1, Fig. 2, Fig. 3 and some measurements of the gap at various magnifications are also provided.

As reported in Table 1 and Table 2, in all groups there were samples where a gap equal to 0 was retrieved, but, in other samples the gap was found to be larger.

**Table 1:**
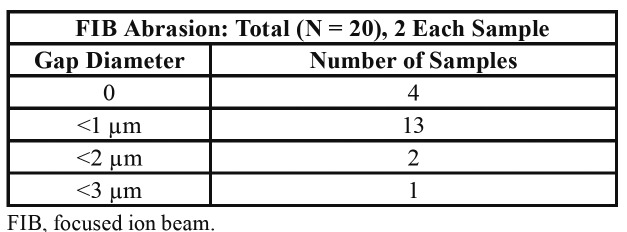
Cross-sections for pure morse cone implants.

**Table 2:**
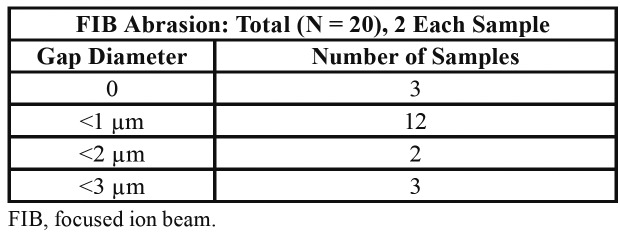
Longitudinal sections for pure morse cone implants.

## Discussion

This study successfully demonstrated that the conical connections analyzed presented a minimal gap at the implant-abutment junction in a static analysis.

The adopted method for measuring this gap was FIB, which represented a reliable method for verifying the effective gap that remains between abutments and fixtures after coupling.

Data from the present study were not intended to give an absolute indication of the advantages or disadvantages of the Morse-type conical connection, but served as an observation through the manipulation of a particular technology, the abrasion of the FIB.

In fact, in previous studies, ordinary observations were carried out by sectioning the samples with microtomes or by electroerosion. Such methods gave rise to considerable artifacts due to the metal surface deformation of the samples occurring in the area where the gap is present and connected with the action of milling or the electroerosion itself (13).

In order to obtain answers regarding the advantages of this type of connection from a microbiological point of view, starting from the assumption that the conical connection may be considered more sTable from a mechanical standpoint than other types of connections, it is necessary to link these studies with those concerning bacterial cultures and with clinical studies on larger sample sizes (14-18).

The present analysis clearly demonstrated the presence of a minimum gap that can promote the access of microorganisms so that it cannot be accepTable to say that the pieces forming the fixture-abutment complex meld together. On the contrary, it can be assumed that, in order to establish real bacterial colonization and multiplication, the bacterial access route must be large enough. In the dataset of this analysis, the width of the gap was less than 1 µm in most cases; this suggests probable unlikelihood for bacterial proliferation.

An interesting observation was the fact that the internal surface of the fixtures adopted in the present study presented a pure Morse cone with a knurling that could leave space for empty areas. On the contrary, the abutment surface is smooth.

Moreover, the Morse-type conical connection guarantees the stability of the interface over time. In case of connection missing the retention itself would be lost unlike the screwed connection in which the 2 parts, although deformed and by masticatory forces, remain connected to each other due to the screw, and are more being subject to colonization by microorganisms (19).

## Conclusions

The observation carried out through the abrasions obtained by FIB SEM allowed to verify in a realistic way the presence of microgaps by eliminating artifacts due to cutting with traditional mechanical tools.

Data reported can confirm a minimal gap at the connection with most of the samples presenting a gap smaller than 1 µm.
